# Supplementary material for: Emergence and Transfer of Plasmid-Harbored rmtB in a Clinical Multidrug-Resistant Pseudomonas aeruginosa Strain
Source: Microorganisms. 2022 Sep 11;10(9):1818. doi: 10.3390/microorganisms10091818 (PMC9500886; doi:10.3390/microorganisms10091818)
Supplement: Supplementary file 1 [file microorganisms-10-01818-s001.zip › Table S1.pdf]

Table S1. Strains and plasmids used in this study

Strains or plasmids	Relevant Characteristics	Source
**Strains**		
P. aeruginosa strain Pa150	Wild-type strain pTJPa150	This study
PAO1-*lacZ*	PAO1 wild-type strain harbored plasmid lacZ	Laboratory storage
PAO1- *lacZ*-pTJPa150	PAO1 wild-type strain harbored plasmid lacZ and plasmid pTJPa150	This study
DH5α		Laboratory storage
**Plasmids**		
pE2031	pUCP24 with promoter deletion	Laboratory storage
pTJPa150	437-kb plasmid isolated from Pa150	This study
