# Supplementary material for: Emergence and Transfer of Plasmid-Harbored rmtB in a Clinical Multidrug-Resistant Pseudomonas aeruginosa Strain
Source: Microorganisms. 2022 Sep 11;10(9):1818. doi: 10.3390/microorganisms10091818 (PMC9500886; doi:10.3390/microorganisms10091818)
Supplement: Supplementary file 1 [file microorganisms-10-01818-s001.zip › Table S2.pdf]

Table S2. Primers used in this study

**Primer**	**Sequence (5'-3')**
rmtB-F	CGGAATTCCACTGATTAAGCATTG
rmtB-R	CGGGATCCTTATCCATTCTTTTTTATC
bla_{TEM}-F	CCTTAAGCTTAGTTTTCGTTC
bla_{TEM}-R	GATGATTTGTCGACTGATTG
parB-F	ATTCATTTGGGACTCGCAGAC
parB-R	GACGCTCCGCAGTAATC

F, forward primer; R, reverse primer.
