# Supplementary material for: Emergence and Transfer of Plasmid-Harbored rmtB in a Clinical Multidrug-Resistant Pseudomonas aeruginosa Strain
Source: Microorganisms. 2022 Sep 11;10(9):1818. doi: 10.3390/microorganisms10091818 (PMC9500886; doi:10.3390/microorganisms10091818)
Supplement: Supplementary file 1 [file microorganisms-10-01818-s001.zip › Table S3.pdf]

Table S3. General features of the *P. aeruginosa* Pa150 genome

Feature	Value for Pa150
Size (bp)	6,582,025
GC content (%)	65.62
Plasmid (bp)	436,716
Tatal no. of coding DNA genes	6,497
No. of CDSs	6,111
No. of RNAs	135
Isolation host	Human diabetic foot
Country	China
