# Supplementary material for: Emergence and Transfer of Plasmid-Harbored rmtB in a Clinical Multidrug-Resistant Pseudomonas aeruginosa Strain
Source: Microorganisms. 2022 Sep 11;10(9):1818. doi: 10.3390/microorganisms10091818 (PMC9500886; doi:10.3390/microorganisms10091818)
Supplement: Supplementary file 1 [file microorganisms-10-01818-s001.zip › Table S4.pdf]

Table S4 Antibiotic resistance genes identified in *P. aeruginosa* Pa150

		**Chromosome**	**Plasmid**
Size (bp)		6,582,025	436,716
	Fosfomycin	*fosA*	
	Amphenicol	*catB7*	*cmlA*
Resistance genes	β-lactam	Bla_{PAO}, bla_{OXA-50}, *ampC*	bla_{TEM}, bla_{OXA-10}
	Aminoglycoside	*aph(3')-IIb*	*aac(3')-IId, rmtB, strA-strB, aac(6')-IIa*
